# Acute Heart Failure and Coronary Blood Flow in ST-Elevation Myocardial Infarction Patients Undergoing Primary Percutaneous Coronary Intervention: An Observational Cohort Study

**DOI:** 10.7759/cureus.50340

**Published:** 2023-12-11

**Authors:** Amr Elkammash, Mohamed Abdelhamid, Mohamed Sobhy, Amr Zaki, Mohamed Sadaka, Oluwamayowa N Omoniyi, Mustafa Alsinan, Rasha M Farahat, Aya Al Sattouf, Khaled Madi

**Affiliations:** 1 Cardiology, Bristol Heart Institute, Bristol, GBR; 2 Cardiology, Alexandria University, Alexandria, EGY; 3 Cardiology, Peterborough City Hospital, Peterborough, GBR; 4 Internal Medicine, Princess of Wales Hospital, Bridgend, GBR; 5 Family Medicine, West Suffolk NHS Foundation Trust, Suffolk, GBR; 6 Medicine, King's College Hospital, London, GBR; 7 Internal Medicine, University Hospitals Dorset NHS Foundation Trust, Bournemouth, GBR

**Keywords:** killip, neurohumoral, perfusion, coronary flow, thrombus, no-reflow, stemi

## Abstract

Background and objective

The Global Registry of Acute Coronary Events (GRACE) study showed poor outcomes in ST-elevation myocardial infarction (STEMI) patients with acute heart failure (AHF) at hospital admission in terms of increased in-hospital and six-month mortality and readmission rates. In this study, we aimed to examine the effects of AHF at the time of admission on the coronary thrombus burden and post-primary percutaneous coronary intervention (PPCI) coronary flow among STEMI patients.

Methods

We conducted a cohort study involving 210 consecutive STEMI patients who presented to a single PPCI centre between June 2016 and January 2017. We classified them into two groups based on their Killip class at the time of presentation to the emergency department: no heart failure (NHF) and AHF groups. The primary outcome was the incidence of Thrombolysis In Myocardial Infarction (TIMI) flow grade of less than 3 in the stented coronary artery in the absence of mechanical obstruction or dissection (also known as no-reflow). The secondary outcome was the presence of a heavy thrombus burden (TIMI grade 4 or 5) at the time of angiography.

Results

The AHF group had a significantly higher incidence of no-reflow than the NHF group (25% vs. 8.4%, p=0.019). However, the prevalence of heavy thrombus burden did not differ significantly between the two groups (50% in the AHF group vs. 43.16% in the NHF group, p=0.557). The multivariable logistic regression analysis showed that AHF was an independent predictor of no-reflow in STEMI patients post-PPCI [Odds ratio (OR): 3.59, 95% confidence interval (CI): 1.09-11.83, p=0.035].

Conclusion

Based on our findings, AHF is associated with an increased risk of no-reflow in STEMI patients post-PPCI, irrespective of the coronary thrombus load.

## Introduction

Acute coronary syndrome (ACS) often leads to acute heart failure (AHF). Simultaneous occurrence of both ACS and AHF is associated with a poor outcome [1,2]. The Global Registry of Acute Coronary Events (GRACE) study, the largest study to investigate AHF in ACS so far, showed that 15.6% of ST-elevation myocardial infarction (STEMI) patients experienced AHF on admission [3]. Subsequently, those patients had a four-fold increase in their in-hospital mortality and a three-fold increase in mortality at six months [3]. In the FINN-AKVA study, the presence of ACS was found to be an independent predictor of 30-day mortality in AHF patients [Hazard ratio (HR): 2, 95% confidence interval (CI): 1.07-3.79, p=0.03] [4]. In the GREAT registry, AHF precipitated by ACS was associated with HR of 1.7 for 90-day mortality compared to AHF without acute coronary event (95% CI: 1.44-1.97, p<0.001) [1].

Heart failure (HF) is linked with the impairment of coronary flow. Multiple mechanisms are involved, including the compression of coronary vessels by left ventricular (LV) pressure, increased reactive oxygen species leading to impaired vasodilatation to nitric oxide, and increased vasoconstrictor mediators including noradrenaline, endothelin, and angiotensin [5]. Animal models have shown that myocardial infarction with subsequent AHF is associated with increased LV end-diastolic wall stress and microvascular perfusion. Such effects can be reversed by unloading the LV by using the Impella CP mechanical circulatory support device [6]. It remains an open question whether the impaired microvascular perfusion in AHF patients can be reproduced in humans with AHF secondary to acute STEMI. The inadequate myocardial perfusion in this patient cohort may explain the poor outcomes demonstrated in the studies above.

The present study aimed to identify the effects of AHF on myocardial perfusion in STEMI patients. We hypothesised that AHF impairs microvascular perfusion in acute myocardial infarction patients irrespective of the size of the coronary thrombus, which can explain the poorer outcomes in this cohort. In light of this, we performed a post-hoc retrospective analysis of the data used in our previously published cohort study that assessed the outcomes in STEMI patients presenting with acute STEMI [7].

## Materials and methods

Study participants

We recruited 230 consecutive STEMI patients presenting to a single primary percutaneous coronary intervention (PPCI) centre between June 2016 and January 2017. They were eligible for PPCI based on the European Society of Cardiology guidelines for acute STEMI [8]. The exclusion criteria included receiving fibrinolysis before presentation, history of percutaneous coronary intervention (PCI) and presentation with stent thrombosis, and patients who presented more than 24 hours from the onset of anginal chest pain, as these factors can affect the thrombus burden in the studied groups. We studied 210 patients using a retrospective cohort design, based on medical records and stored angiographic data. We excluded 20 patients (five patients who received fibrinolysis, 10 who presented with acute stent thrombosis after a previous PCI, and five who presented more than 24 hours after the onset of pain). We stratified the candidates into two groups based on their Killip class at the time of presentation to the emergency department: the patients in Killip class I [no heart failure (NHF) group] and those in Killip class II-IV (AHF group) [9].

Primary and secondary outcomes

The primary outcome was impaired coronary flow [Thrombolysis In Myocardial Infarction (TIMI) flow grade of less than 3 despite the absence of mechanical obstruction or dissection (also known as no-reflow)] (Table 1) [10]. The secondary outcome was the angiographic heavy thrombus burden (TIMI thrombus grade 4 or 5) (Table 2) [11].

**Table 1:** TIMI blood flow grades TIMI: Thrombolysis in Myocardial Infarction

Grade	Definition
Grade 0	No flow at all after the obstruction point
Grade 1	The contrast material flows beyond the area of obstruction but fails to opacify the entire artery
Grade 2	Opacification of the entire artery distal to the occlusion site but at a slower rate than normal
Grade 3	Normal coronary flow

**Table 2:** TIMI thrombus grades TIMI: Thrombolysis in Myocardial Infarction

Grade	Definition
Grade 0	No angiographic evidence of thrombus
Grade 1	Angiographic features suggestive of thrombus (decreased contrast density, haziness of contrast, irregular lesion contour, a smooth convex meniscus at the site of a total occlusion, suggestive, but not firmly diagnostic of thrombus)
Grade 2	Definite thrombus present in multiple angiographic projections (marked irregular lesion contour with a significant filling defect—the greatest dimension of thrombus is <1/2 vessel diameter)
Grade 3	Definite thrombus appears in multiple angiographic views (greatest dimension from >1/2 to <2 vessel diameters)
Grade 4	Definite large-size thrombus present (greatest dimension >2 vessel diameters)
Grade 5	Definite complete thrombotic occlusion of a vessel (a convex margin that stains with contrast, persisting for several cardiac cycles)

Assessment of angiographic findings

Two experienced interventional cardiologists with at least five years of experience in PPCI procedures assessed the angiographic thrombus burden and post-PPCI outcomes in the studied groups while blinded to the patient characteristics.

Statistical analysis

We used the IBM SPSS Statistics software version 22.0 (IBM Corp., Armonk, NY) to run the statistical analysis. Quantitative data were expressed using mean and standard deviation (SD), while qualitative data were expressed in frequency and percentage. Qualitative data were analysed using the Chi-square test. Non-normally distributed quantitative data were analysed using the Mann-Whitney U test to compare the two groups. We used a multivariable logistic regression analysis to study the relationship between AHF and post-PPCI coronary flow, among other cardiovascular risk factors, namely diabetes, hypertension, smoking, dyslipidemia, and old myocardial infarction. The p-value was assumed to be significant at 0.05.

## Results

Twenty patients (9.5%) presented with AHF. The baseline characteristics of the studied groups are shown in Table 3. The two studied groups were well matched across the different cardiovascular risk factors, including diabetes mellitus, hypertension, smoking, dyslipidemia, as well as previous myocardial infarction and cerebrovascular events.

**Table 3:** Baseline characteristics of the studied groups NHF: no heart failure; AHF: acute heart failure; IDDM: insulin-dependent diabetes mellitus; NIDDM: non-insulin-dependent diabetes mellitus; MI: myocardial infarction; CVA: cerebrovascular accident; SD: standard deviation

Parameter	NHF group	AHF group	P-value
Number of patients (%)	190 (90.5%)	20 (9.5%)	-
Age, years, mean ±SD	54.99 ±11.3	59.95 ±7.62	-
Sex, n (%)			
Male	170 (89%)	17 (85%)	0.24
Female	20 (11%)	3 (15%)	
Hypertension, n (%)	73 (38%)	8 (40%)	0.98
IDDM, n (%)	6 (3%)	0 (0%)	0.41
NIDDM, n (%)	70 (37%)	10 (50%)	0.33
Smoking, n (%)	113 (59%)	13 (65%)	0.83
Dyslipidemia, n (%)	112 (59%)	14 (70%)	0.49
Previous MI, n (%)	7(3.7%)	1 (5%)	0.81
Previous CVA, n (%)	1 (0.5%)	0 (0%)	0.74

Primary outcome

**Figure 1:**
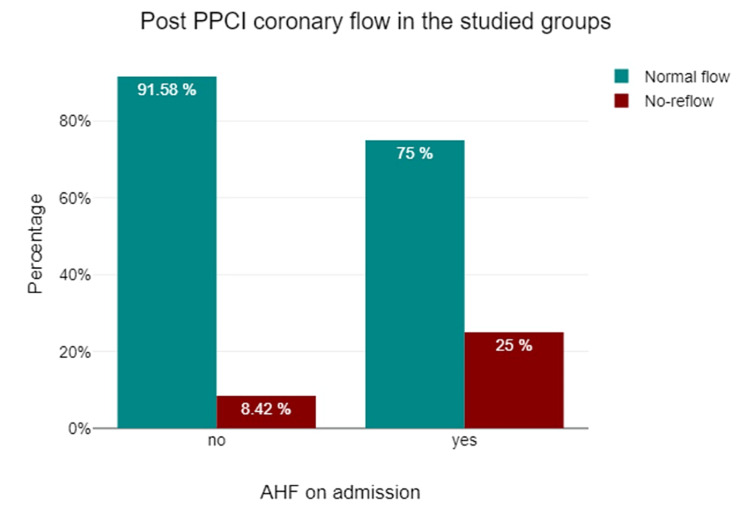
The incidence of no-reflow in STEMI patients with and without AHF on admission STEMI: ST-elevation myocardial infarction; AHF: acute heart failure; PPCI: primary percutaneous coronary intervention

To investigate whether the incidence of the no-reflow was higher in the AHF group, we used the Chi-square test. No-reflow was significantly more common in the AHF group (25% vs. 8.4%, p=0.019) (Figure 1, Table 4). The multivariable logistic regression analysis showed that AHF on admission was an independent predictor of no-reflow in STEMI patients after PPCI [odds ratio (OR): 3.59, 95% CI: 1.09-11.83, p=0.035) (Table 5).

**Table 4:** The distribution of different outcomes in the studied groups NHF: no heart failure; AHF: acute heart failure

Parameter	NHF group (n=190)	AHF group (n=20)	P-value
Heavy thrombus burden, n (%)	82 (43.16%)	10 (50%)	0.557
No-reflow, n (%)	16 (8.4%)	5 (25%)	0.019

**Table 5:** Logistic regression model for the prediction of no-reflow in STEMI patients with AHF STEMI: ST-elevation myocardial infarction; AHF: acute heart failure; OR: odds ratio; CI: confidence interval

Variable	Univariable model			Multivariable model		
	OR	95% CI	P-value	OR	95% CI	P-value
Smoking	1.76	0.65-4.73	0.27	2	0.71-5.63	0.19
Hypertension	1.51	0.61-3.74	0.37	1.4	0.5-3.95	0.52
Diabetes	0.51	0.21-1.26	0.15	1.66	0.59-4.68	0.34
Hyperlipidemia	0.58	0.24-1.45	0.25	0.7	0.26-1.88	0.47
Heavy thrombus burden	2.85	1.1-7.38	0.031	2.64	0.98-7.11	0.06
Acute heart failure	3.62	1.17-11.27	0.026	3.59	1.09-11.83	0.035

Secondary outcome

**Figure 2:**
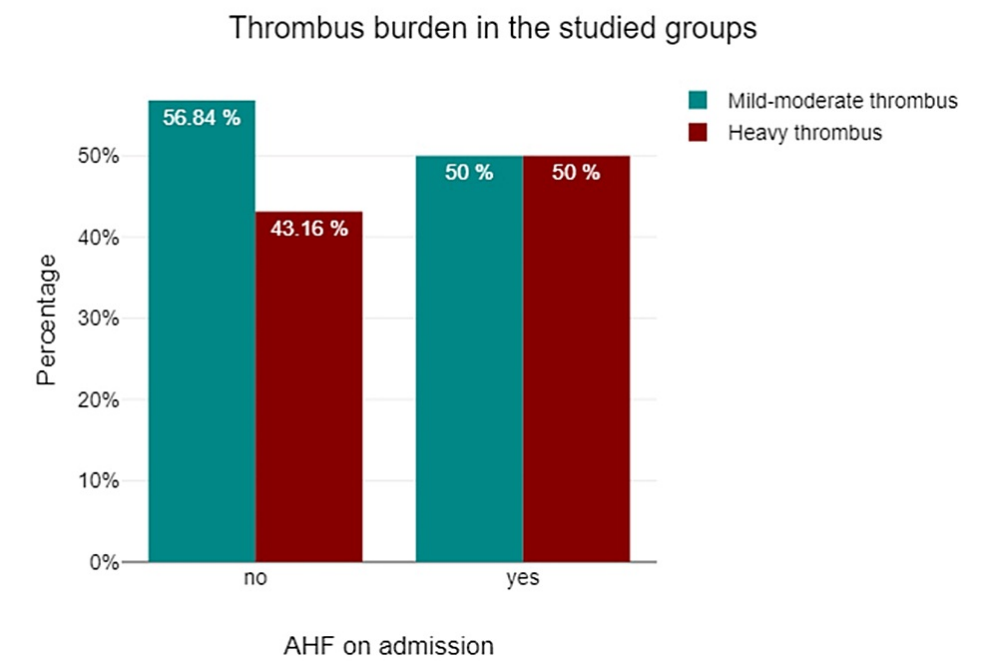
The angiographic thrombus burden in STEMI patients with and without AHF on admission STEMI: ST-elevation myocardial infarction; AHF: acute heart failure

We used the Chi-square test to investigate the association between the AHF and the presence of heavy thrombus burden in the two groups. The prevalence of high thrombus burden did not differ significantly between the NHF and AHF groups (43.16% vs. 50%, p=0.557) (Table 4, Figure 2).

## Discussion

Our study illustrated the association between AHF and impaired coronary blood flow in STEMI patients, irrespective of the coronary thrombus load on angiography. The impaired coronary flow increases in-hospital and long-term morbidity and mortality in these patients [12,13]. Therefore, early reperfusion in STEMI is necessary to prevent AHF development and its serious effects on coronary flow and the patient’s prognosis. Our results shed light on the worse outcomes in ACS patients who develop AHF.

No-reflow in STEMI patients with AHF has a few explanations. Firstly, HF causes coronary microvascular dysfunction even without coronary artery disease (CAD) [14]. Animal studies have shown that HF impairs endothelial-derived nitric oxide formation [15] and parasympathetically-induced coronary vasodilation [16]. HF also induces microcirculation vasoconstriction by neurohumoral activation mediators (norepinephrine, epinephrine, and endothelin) [5]. Secondly, as demonstrated in dog models, coronary microembolisation of atheromatous debris during PCI causes further endothelial dysfunction and impairment of the coronary reserve [17].

Tanboga et al. [18] have studied the prevalence of HF on admission among STEMI patients with and without heavy coronary thrombus burden. The study found that those with a heavy thrombus burden had a higher prevalence of HF on admission. The study also revealed a higher incidence of no-reflow in the HF group, which was proposed to be secondary to the higher incidence of distal embolisation in HF patients with a high thrombus burden. However, in our study, the increased no-reflow in the HF group was independent of the heavy thrombus burden, implicating neurohumoral mechanisms as a cause of the impaired perfusion. This theory has been further reinforced by the improvement in myocardial perfusion after unloading LV with Impella CP in animal models [6].

Strengths and limitations

Our study contributes to the body of evidence on the relationship between AHF, coronary thrombus load, and post-PPCI coronary flow in STEMI patients. The results support the theory of non-thrombus-related mechanisms causing impaired coronary flow in STEMI patients with AHF. The two studied groups were matched in terms of various cardiovascular risk factors. However, such significant results came at the cost of a few limitations. Firstly, the study was conducted at a single center and included patients from a single ethnic group, impairing the generalisability of the results. Secondly, due to the observational and retrospective nature of the study, we could not control other procedural factors that may have affected the final TIMI flow grade, such as the use of glycoprotein IIb/IIIa inhibitors, the type of P2Y12 inhibitor used, the length of the stented lesion, and the type of drug-eluting stent used. Lastly, HF was identified by clinical signs without using laboratory markers [e.g., N-terminal pro-B-type natriuretic peptide (NT-proBNP)] or invasive measures (e.g., LV filling pressures). Hence, we may have missed patients with subclinical HF in our analysis. Nevertheless, depending merely on clinical signs makes our results applicable where these investigations are not available. Future research involving prompt detection of HF using laboratory markers (e.g., NT-proBNP) and studying long-term effects of early HF treatment is needed to gain deeper insights into the impact of AHF on the prognosis of STEMI patients.

## Conclusions

STEMI patients with AHF on admission can have a higher risk of impaired coronary flow post-PPCI, irrespective of the coronary thrombus load. The inadequate flow increases in-hospital and long-term morbidity and mortality in these patients. Therefore, the treating team should adequately communicate such risks to these patients and arrange all the necessary facilities to manage the possible complications.
